# Musculo-Immuno-Nutritional Score as a Prognostic Marker in Patients with Interstitial Pneumonia Awaiting Lung Transplantation

**DOI:** 10.5761/atcs.oa.25-00067

**Published:** 2025-06-05

**Authors:** Gouji Toyokawa, Miho Yamaguchi, Takafumi Yamaya, Mitsuaki Kawashima, Chihiro Konoeda, Mototsugu Shimokawa, Masaaki Sato

**Affiliations:** 1 Department of Thoracic Surgery, The University of Tokyo Hospital, Bunkyo-Ku, Tokyo, Japan; 2 Department of Surgery and Science, Graduate School of Medical Sciences, Kyushu University, Fukuoka, Fukuoka, Japan; 3 Department of Biostatistics, Graduate School of Medicine, Yamaguchi University, Ube, Yamaguchi, Japan

**Keywords:** lung transplantation, musculo-immuno-nutritional score, waitlist mortality

## Abstract

**Purpose:** This study evaluated the prognostic significance of the controlling nutritional status/creatine kinase score (CNKS), a composite index derived from the controlling nutritional status (CONUT) score and creatine kinase (CK) level, in patients with interstitial pneumonia awaiting lung transplantation (LT).

**Methods:** We retrospectively analyzed 202 patients with interstitial pneumonia who were registered for LT between January 2014 and July 2023. CNKS was calculated using CK levels and the CONUT (derived from albumin level, lymphocyte count, and cholesterol level).

**Results:** Among the 202 patients, 130 (64.4%) were alive, while 72 (35.6%) had died at the time of analysis. Among the surviving patients, 79 (39.1%) underwent cadaveric LT, and 51 (25.2%) remained on the waiting list. A high CNKS (n = 72 [35.6%]) was significantly associated with a lower body mass index (*P* <0.001), a shorter 6-minute walk distance (*P* <0.001), and lower forced vital capacity (*P* = 0.006) compared with a low CNKS (n = 130 [64.4%]). The results of the multivariate analysis showed that CNKS was a significant independent prognostic factor for survival during the waiting period (*P* = 0.031).

**Conclusion:** CNKS represents a promising prognostic marker, potentially useful in selecting lung transplant candidates and guiding nutritional and rehabilitative interventions during the pretransplant period.

## Introduction

Lung transplantation (LT) is considered for patients with end-stage lung diseases, such as interstitial pneumonia (IP), pulmonary arterial hypertension, chronic obstructive pulmonary disease, and allogeneic diseases, such as graft-versus-host disease after hematopoietic stem cell transplantation and chronic lung allograft dysfunction, as well as suppurative diseases. However, not all patients with end-stage lung disease can successfully undergo LT. For instance, in Japan, the median waiting time for cadaveric LT is around 700 days even after the revision of the organ transplant law, with the mortality rate in patients awaiting LT being as high as 33.1%.^1)^ To shorten the waiting period and consequently reduce the waiting list mortality rate, it is important not only to establish an optimum allocation system specific to each country but also to manage factors that negatively affect mortality (e.g., disease severity, nutritional status, and muscle mass) in patients awaiting LT.

Nutritional status is associated with postoperative mortality after LT and waitlist mortality in patients registered for LT.^2,3)^ In addition, recent studies have revealed the prognostic role of skeletal muscle mass in both postoperative mortality after LT and waitlist mortality in patients awaiting LT.^4,5)^ Thus, both nutritional status and muscle mass are important in patients registered for LT; however, there are no markers that represent both of them in such patients. We hypothesized that such markers would be helpful in improving the management of patients awaiting LT and in predicting their mortality during the waiting period.

Previously, we reported the prognostic role of a musculo-immuno-nutrition score, called the controlling nutritional status/creatine kinase (CK) score (CNKS), in patients with non-small cell lung cancer (NSCLC).^6)^ Namely, CNKS is formed from a combination of the “controlling nutritional status” (CONUT; calculated by measuring the serum albumin level, lymphocyte count, and cholesterol level) and CK. In that study, CK level was shown to be significantly associated with muscle mass of Th12, suggesting that CK may serve as a surrogate marker for skeletal muscle mass, and patients with high CNKS (indicating low nutrition status and low CK, a surrogate of low muscle mass) showed significantly poor prognosis than those with low CNKS in NSCLC.^6)^ Based on the above, CNKS might be a suitable marker to predict the mortality of patients awaiting LT.

In the current study, we investigated the relationship between CNKS and patient characteristics, such as age, sex, 6-minute walk distance, oxygen supplementation, pulmonary function, and blood test results in patients awaiting LT, focusing especially on those with IP. The prognostic role of CNKS in this patient group was also examined.

## Materials and Methods

### Patients

In total, 398 patients were registered for LT at the University of Tokyo Hospital between January 2014 and July 2023. The patient characteristics are shown in **Supplementary Table 1**. The patient breakdown by disease type was as follows: IP (n = 223 [56.0%]), pulmonary vascular disease (n = 72 [18.1%]), obstructive pulmonary disease (n = 42 [10.6%]), suppurative pulmonary disease (n = 29 [7.3%]), and allogeneic disease (n = 32 [8.0%]). Until the July 2024 data cut-off point, data relating to LT, waiting, and death were collected (**Supplementary Fig. 1A**). Given that patients with IP had the worst prognosis (**Supplementary Fig. 1B**), the investigation of the prognostic significance of CNKS was prioritized for this patient group.

### Calculation of CONUT and CNKS

CONUT was calculated as the sum of albumin level, lymphocyte count, and cholesterol level measurement (**Table 1**),^7)^ and each variable was scored as follows: albumin level (g/dL): ≥3.50: 0, 3.00–3.49: 2, 2.50–2.99: 4, <2.50: 6; cholesterol level (mg/dL): ≥180: 0, 140–179: 1, 100–139: 2, <100: 3; lymphocyte count (/mm^3^): ≥1600: 0, 1200–1599: 1800–1199: 2, <800: 3. CNKS was obtained from the sum of the CONUT and CK level values (**Table 1**),^6)^ and CK level was scored as follows: CK level (mg/dL): male: ≥62: 0, <62: 2; female: ≥45: 0, <45: 2.

**Table 1:** CONUT and CNKS score

Factor	Range	Score
Albumin level (g/dL)	≥3.50	0
	3.00–3.49	2
	2.50–2.99	4
	<2.50	6
Cholesterol level (mg/dL)	≥180	0
	140–179	1
	100–139	2
	<100	3
Lymphocyte count (/mm^3^)	≥1600	0
	1200–1599	1
	800–1199	2
	<800	3
Creatine kinase level (mg/dL)	Male: ≥62	0
	Female: ≥45	
	Male: <62	2
	Female: <45	

CONUT score: albumin score + cholesterol score + lymphocyte count score; CNKS score: CONUT score + creatine kinase score.

CONUT: controlling nutritional status; CNKS: controlling nutritional status/creatine kinase score

### Statistical analysis

Categorical variables were summarized as numbers and percentages, while continuous variables were presented as mean or median values depending on their distribution. Overall survival was calculated from the date of registration for LT (i.e., registration on the Japan Organ Transplant Network for brain-dead LT or at our institution for living-donor LT) to the date of death from any cause. Patients who underwent LT were censored at the time of the operation. Student's t-test and the Mann–Whitney test (for data that were not normally distributed) were used to analyze the associations between CNKS and continuous data. The chi-squared test was used to analyze the associations between CNKS and categorical variables. Survival probabilities were estimated by using the Kaplan–Meier method, and differences in the survival probabilities were analyzed using the log-rank test. Risk factors for waiting mortality were assessed using a Cox proportional hazards model. Differences were considered to be statistically significant when the *P*-value was less than 0.05. All analyses were performed using JMP^®^ 18.0 (SAS Institute, Cary, NC, USA) and Prism 8.0 (GraphPad Software, San Diego, CA, USA) software.

## Results

### Patient characteristics

**Fig. 1:**
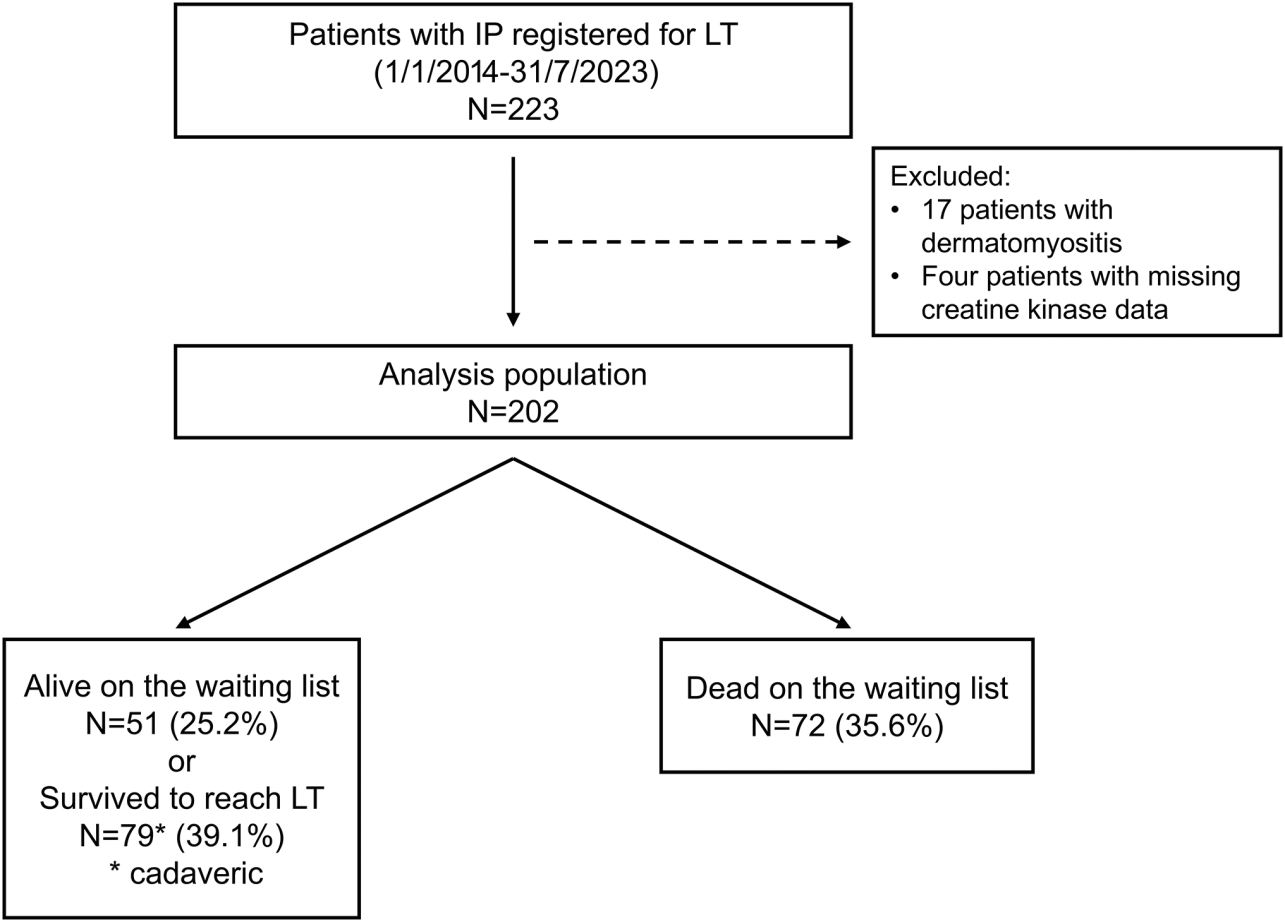
Study flowchart. The analysis population consisted of 202 patients with IP. IP: interstitial pneumonia; LT: lung transplantation

The study flow diagram is shown in **Fig. 1**. Among the 223 patients with IP, 17 were excluded due to having dermatomyositis, because CK levels often reflect disease activity in such patients, making it difficult to distinguish whether they are indicative of muscle mass or disease activity. In addition, 4 patients were excluded due to missing CK data. As a result, 202 patients with IP were included in the final analysis. Among them, 130 patients (64.4%) were either alive on the waiting list, or survived to reach LT, while 72 (35.6%) had died on the waiting list. Of the 130 surviving patients, 79 (39.1%) underwent cadaveric LT, and 51 (25.2%) remained on the waitlist for LT.

The characteristics of the 202 patients with IP at the time of registration for LT are shown in **Table 2**. The median length of follow-up was 428.5 days for these 202 patients. The median age was 52 years (range: 6–60), 68 patients (33.7%) were female, and 123 patients (60.9%) had a history of smoking. The median body mass index (BMI) and 6-minute walk distance values were 22.0 kg/m^2^ (range: 10.5–34.9) and 335 m (range: 10–660), respectively. The breakdown of patients by IP type was as follows: idiopathic pulmonary fibrosis (n = 51 [25.3%]), idiopathic pleuroparenchymal fibroelastosis (n = 34 [16.8%]), collagen disease-associated IP (n = 37 [18.3%]), and others (n = 80 [39.6%]). The median percentage of forced vital capacity (FVC) was 48.1% (range: 12.4–125.1).

**Table 2:** Characteristics of patients with interstitial pneumonia at the time of registration for lung transplantation

Demographics	N (%)
Age at registration (years)*	52 (6–60)
Sex	
Female	68 (33.7%)
Male	134 (66.3%)
Smoking history	
Never	79 (39.1%)
Ex- or current	123 (60.9%)
BMI (kg/m^2^)*	
Mean ± SD	21.9 ± 4.8
6-minute walk distance (m)*	
Median (range)	335 (10–660)
Blood type	
A	84 (41.6%)
O	52 (25.7%)
B	49 (24.3%)
AB	17 (8.4%)
Type of IP	
IPF	51 (25.3%)
IPPFE	34 (16.8%)
Collagen disease-associated	37 (18.3%)
Other	80 (39.6%)
Oxygen at rest	103 (51.0%)
Oxygen on exertion	152 (75.2%)
Use of steroid	108 (53.5%)
Use of antifibrotic agents	109 (54.0%)
Mean PAP (mmHg)*	
Median (range)	18 (7–68)
Pulmonary hypertension	41 (20.7%)
Lung function*	
FVC (L), median (range)	1.9 (0.4–4.4)
%FVC (%), median (range)	48.1 (12.4–125.1)
FEV1.0 (L), median (range)	1.6 (0.4–3.3)
FEV1.0/FVC (%), median (range)	88.2 (28.1–100)
%DLCO (%), mean ± SD	41.6 ± 14.5
%DLCO/VA (%), mean ± SD	72.9 ± 24.8
Laboratory data*	
pO_2_ (mmHg), median (range)	76.6 (38.9–179)
pCO_2_ (mmHg), median (range)	41.5 (28.7–97)
Albumin (g/dL), median (range)	4.0 (2.7–4.9)
Creatine kinase (IU/L), median (range)	72 (8–334)
Total cholesterol (mg/dL), median (range)	195 (117–357)
Lymphocyte count (10^3^/µL), median (range)	1.5 (0.2–4.3)
Neutrophil count (10^3^/µL), median (range)	5.3 (0.8–17.2)
CRP (mg/dL), median (range)	0.23 (0.02–7.26)
KL-6 (U/mL), median (range)	927 (14–12153)
BNP (pg/mL), median (range)	14.6 (2.0–1704)

*Median value (range).

BMI: body mass index; BNP: brain natriuretic peptide; CRP: C-reactive protein; DLCO: diffusing capacity of the lungs for carbon monoxide; DLCO/VA: DLCO divided by the alveolar volume; FEV1.0: forced expiratory volume in 1 second; FVC: forced vital capacity; IP: interstitial pneumonia; IPF: idiopathic pulmonary fibrosis; IPPFE: idiopathic pleuroparenchymal fibroelastosis; PAP: pulmonary arterial pressure; KL-6: Krebs von den Lungen-6; pCO_2_: partial pressure of carbon dioxide; pO_2_: partial pressure of oxygen; SD: standard deviation

### Distribution of CNKS and its cut-off

The median CNKS was 2 (range: 0–14) as shown in **Supplementary Fig. 2A**. A receiver-operating characteristic (ROC) curve for overall mortality revealed that the optimal cut-off value for CNKS was also 2 (low: ≤2; high: ≥3), with a sensitivity, specificity, and area under the ROC curve (AUC) of 72.3%, 50.0%, and 0.625, respectively (**Supplementary Fig. 2B**). The cut-off value for CONUT was 1, with a sensitivity, specificity, and AUC of 63.1%, 56.9%, and 0.621, respectively (data not shown).

### Association between CNKS and patient characteristics

Next, we divided the 202 patients with IP into the CNKS-high (≥3; n = 72 [35.6%]) and CNKS-low (≤2; n = 130 [64.4%]) groups according to the CNKS cut-off value. Patients in the CNKS-high group had a significantly lower BMI (*P* <0.001), a shorter 6-minute walk distance (*P* <0.001), required oxygen supplementation (*P* <0.001), steroid use (*P* <0.001), did not use antifibrotic agents (*P* = 0.004), and had a higher mean pulmonary arterial pressure (*P* = 0.009), lower FVC (*P* = 0.006), lower forced expiratory volume in 1 second (*P* = 0.013), higher pCO_2_ (*P* = 0.039), higher C-reactive protein (CRP) level (*P* <0.001), and higher brain natriuretic peptide level (*P* <0.001) than those in the CNKS-low group (**Table 3**).

**Table 3:** Characteristics of CNKS-high/-low patients with interstitial pneumonia at the time of registration for lung transplantation

Demographic	CNKS-low group (≤2; N = 130 [64.3%])	CNKS-high group (≥3; N = 72 [35.7%])	*P-*value
Age (years), median (range)	53 (6–60)	50 (10–60)	0.171
Sex			
Female	46 (35.4%)	22 (30.6%)	0.487
Male	84 (64.6%)	50 (69.4%)	
Smoking history			
Never	50 (38.5%)	29 (40.3%)	0.800
Ex- or current	80 (61.5%)	43 (59.7%)	
BMI (kg/m^2^), mean ± SD	22.7 ± 0.4	20.3 ± 0.5	<0.001
Blood type			
A	55 (42.3%)	29 (40.3%)	0.886
O	32 (24.6%)	20 (27.8%)	
B	33 (25.4%)	16 (22.2%)	
AB	10 (7.7%)	7 (9.7%)	
Type of IP			
IPF	37 (28.5%)	14 (19.4%)	0.237
IPPFE	22 (16.9%)	12 (16.7%)	
CD-associated	19 (14.6%)	18 (25.0%)	
Others	52 (40.0%)	28 (38.9%)	
6-min walk distance (m), median (range)	360 (0–660)	298 (20–500)	<0.001
Oxygen level at rest			
No	76 (58.5%)	23 (31.9%)	<0.001
Yes	54 (41.5%)	49 (68.1%)	
Oxygen level on exertion			
No	42 (32.3%)	8 (11.1%)	<0.001
Yes	88 (67.7%)	64 (88.9%)	
Steroid use			
No	72 (55.4%)	22 (30.6%)	<0.001
Yes	58 (44.6%)	50 (69.4%)	
Antifibrotic agent use			
No	50 (38.5%)	43 (59.7%)	0.004
Yes	80 (61.5%)	29 (40.3%)	
Mean PAP (mmHg), median (range)*	17 (7–68)	20 (7–50)	0.009
Pulmonary hypertension*			
No	108 (85.0%)	49 (69.0%)	0.008
Yes	19 (15.0%)	22 (30.1%)	
FVC (L), median (range)	2.00 (0.85–4.39)	1.50 (0.40–3.76)	0.006
%FVC (%), median (range)	49.5 (17.7–125.1)	41.1 (12.4–85.6)	0.003
FEV1.0 (L), median (range)	1.75 (0.81–3.01)	1.35 (0.40–3.34)	0.013
FEV1.0/FVC (%), median (range)	87.7 (38.5–100)	89.1 (28.1–100)	0.375
%DLCO (%), mean ± SD	44.0 ± 1.3	36.2 ± 2.0	0.001
%DLCO/VA (%), mean ± SD	77.8 ± 2.3	61.8 ± 3.4	<0.001
pO_2_ (mmHg), median (range)	76.7 (41.0–179)	74.6 (38.9–170)	0.245
pCO_2_ (mmHg), median (range)	41.1 (28.7–97)	43.1 (30.9–74.2)	0.039
CRP level (mg/dL), median (range)	0.18 (0.02–2.45)	0.32 (0.02–7.26)	<0.001
KL-6 (U/mL), median (range)	930 (14.2–6703)	926 (200–12153)	0.414
BNP level (pg/mL), median (range)	12.6 (2.0–660.7)	20.2 (2.9–1704.3)	<0.001

*Data were missing from 4 patients.

BMI: body mass index; BNP: brain natriuretic peptide; CNKS: controlling nutritional status/creatine kinase score; CRP: C-reactive protein; DLCO: diffusing capacity of the lungs for carbon monoxide; DLCO/VA: DLCO divided by the alveolar volume; FEV1.0: forced expiratory volume in 1 second; FVC: forced vital capacity; IP: interstitial pneumonia; IPF: idiopathic pulmonary fibrosis; IPPFE: idiopathic pleuroparenchymal fibroelastosis; KL-6: Krebs von den Lungen-6; PAP: pulmonary arterial pressure; pCO_2_: partial pressure of carbon dioxide; pO_2_: partial pressure of oxygen; SD: standard deviation

### Analysis of risk factors for waiting mortality

**Fig. 2:**
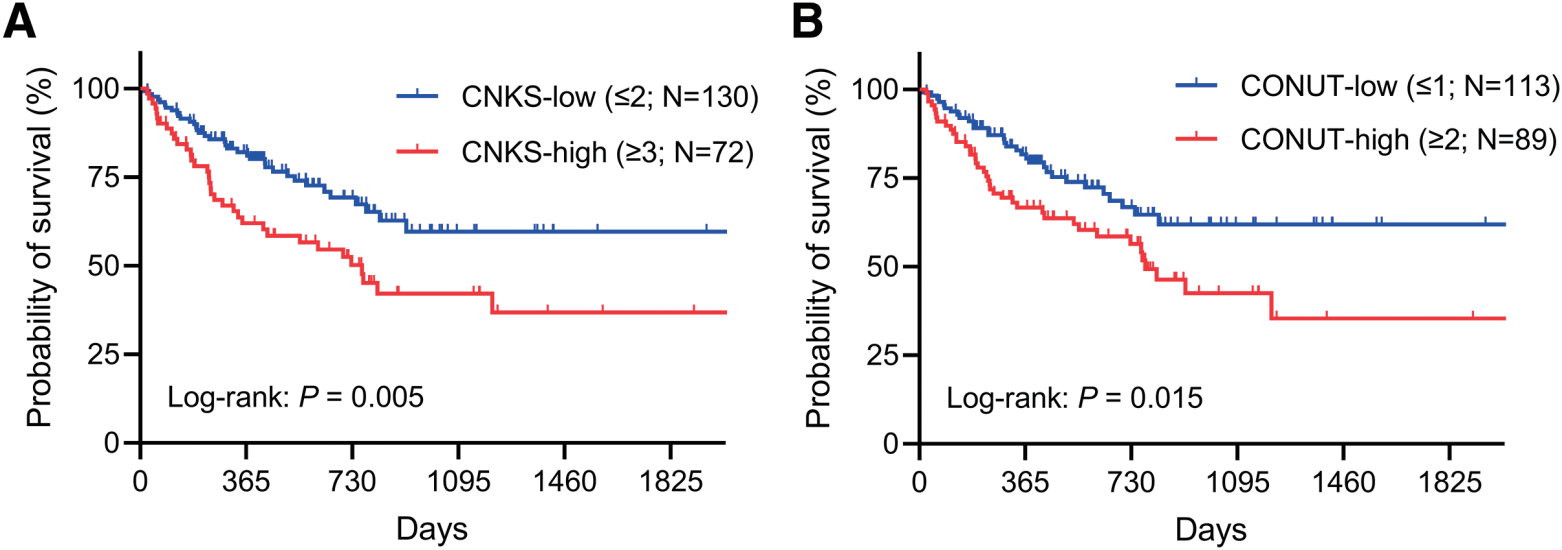
Overall survival curves of 202 patients with IP after they had registered for LT according to (**A**) CNKS or (**B**) CONUT. CNKS: controlling nutritional status/creatine kinase score; CONUT: controlling nutritional status; IP: interstitial pneumonia; LT: lung transplantation

Patients in the CNKS-high group had a significantly shorter survival time than those in the CNKS-low group (*P* = 0.005; **Fig. 2A**). Similarly, patients in the CONUT-high group had a significantly shorter survival time than those in the CONUT-low group (*P* = 0.015; **Fig. 2B**). Multivariate analysis showed that CNKS (*P* = 0.031), age (*P* = 0.014), BMI (*P* = 0.017), and 6-minute walk distance (*P* <0.001) were significant independent prognostic factors for survival during the waiting period (**Table 4**).

**Table 4:** Results of the univariate and multivariate analyses of mortality in the cohort of patients with IP awaiting lung transplantation

Variables	Univariate analysis			Multivariate analysis		
	HR	95% CI	*P*-value	HR	95% CI	*P*-value
Age (>52/≤52)	1.269	0.797–2.018	0.315	1.947	1.148–3.302	0.014
Sex (female/male)	0.892	0.549–1.451	0.646	0.811	0.466–1.409	0.457
Smoking history (never/ex- or current)	0.948	0.588–1.529	0.827	0.898	0.511–1.577	0.708
BMI (>22.0/≤22.0 kg/m^2^)	0.572	0.362–0.914	0.019	0.525	0.309–0.891	0.017
6-minute walk distance (>335/≤335 m)	0.330	0.198–0.552	<0.001	0.355	0.207–0.610	<0.001
Oxygen level at rest (yes/no)	1.675	1.053–2.666	0.030	1.290	0.768–2.165	0.336
CNKS (high [≥3]/low [≤2])	1.968	1.239–3.127	0.004	1.723	1.050–2.825	0.031

BMI: body mass index; CI: confidence interval; CNKS: controlling nutritional status/creatine kinase score; HR: hazard ratio; IP: interstitial pneumonia

## Discussion

In this study, CNKS was shown to be an independent prognostic predictor in patients with IP awaiting LT. The importance of nutrition-related factors in predicting the mortality of patients with IP while awaiting LT has been reported.^8)^ In addition, recent studies have revealed the prognostic role of skeletal muscle mass for both postoperative mortality after LT and waitlist mortality in patients awaiting LT.^4,5)^ However, to the best of our knowledge, this study is the first to clarify the significance of using a score that combines nutrition-related factors and CK level (as a possible surrogate marker for muscle mass) to predict waitlist mortality in patients with IP. Measuring CK level is easier, less invasive, and more cost-effective than measuring skeletal muscle mass; moreover, it is routinely performed in standard clinical practice.

It has been reported that CONUT is important in predicting survival in various diseases. For instance, a high CONUT was associated with a worse prognosis in patients with NSCLC who underwent surgical resection.^9,10)^ In addition, a recent study has shown that CONUT was significantly associated with mortality in patients with idiopathic pulmonary fibrosis.^11)^ Based on the hypothesis that adding CK, which might serve as a surrogate for muscle mass), to CONUT is superior to CONUT alone in predicting survival in lung cancer, we previously created CNKS and demonstrated its superiority to CONUT as a prognostic factor in NSCLC.^6)^ Also, in the present study, CNKS was superior to CONUT in stratifying the survival curve in patients with IP who were registered for LT (**Fig. 2**). However, our analysis of the full cohort of patients awaiting LT did not identify CNKS as a prognostic factor (**Supplementary Fig. 3**), suggesting that its prognostic impact may differ depending on the disease.

The present study highlighted the association between CNKS and several clinicopathological factors. Specifically, high CNKS values were significantly associated with a lower BMI, a shorter 6-minute walk distance, lower pulmonary function, higher mean pulmonary arterial pressure, and higher CRP level compared to low CNKS values, suggesting that CNKS can be an indicator of the patient's general status and can be used to monitor the disease progression. In addition, the association between CNKS and steroid use might be due to steroid myopathy, as well as the reduction in lymphocyte counts caused by long-term steroid use, although this remains a hypothesis.

The role of CNKS as an independent prognostic predictor in patients with IP has several important clinical implications. First, patients with high CNKS at the time of registration for LT may not be suitable candidates for LT. In the International Society for Heart and Lung Transplantation consensus document for the selection of LT candidates, nutritional status and frailty were reported as factors that could lead to poor outcomes after LT.^12)^ Second, it may be possible to reduce the waiting list mortality of patients with high CNKS at the time of LT registration by improving muscle mass and nutritional status. In fact, preoperative rehabilitation has been reported to be safe and effective at improving the 6-minute walk distance, quality of life, and mood status, while simultaneously reducing dyspnea in patients awaiting LT.^13)^ The leucine metabolite β-hydroxy-β-methylbutyrate (HMB) can be used to reduce protein catabolism and promote skeletal muscle hypertrophy.^14)^ A double-blinded study showed that a combination of HMB administration and nutritional counseling improved muscle mass and reduced the frailty index of patients awaiting liver transplantation.^15)^ Third, CNKS can also be used as an indicator for early transplant organ allocation. For instance, the United States uses a lung allocation system to reduce waiting list mortality.^16)^ In Japan, however, LT is currently performed on a first-come, first-served basis. Parameters like CNKS would help identify patients most likely to benefit from LT.

This study is associated with some limitations. First, although our sample size was relatively large (202 patients with IP), the retrospective and single-center nature of this study may have resulted in selection bias. Second, the relationship between skeletal muscle mass and CK level was not analyzed in this study and will need to be explored in future studies. Third, the fact that all the patients who participated in this study were Japanese may limit study's generalizability because the physical and nutritional characteristics of patients differ among countries. Fourth, since patients who received LT were censored at the time of transplantation, this could introduce informative censoring, potentially leading to bias in the survival analysis. Fifth, an ROC-AUC of 0.625 indicates only modest predictive power, and the ROC analysis was not internally validated due to the relatively small number of patients included in this study. Future studies with a larger number of patients are warranted to perform internal validation and strengthen confidence in the cut-off point of CNKS. Sixth, no patients with IP underwent living-donor LT, and therefore, a subgroup analysis by type of LT was not performed. Future studies are warranted to clarify the significance of CNKS in patients with IP who receive living-donor LT.

## Conclusion

In conclusion, our results suggest that CNKS at the time of registration is an independent prognostic factor for waiting list mortality and can be used to adequately assess the condition of patients awaiting LT. As such, CNKS may represent a promising new prognostic marker for the selection of candidates for LT.

## Acknowledgments

We thank Edanz (https://jp.edanz.com/ac) for editing the English text of a draft of this manuscript.

## Declarations

### Ethics approval and consent for publication

This retrospective, descriptive, exploratory study of an ongoing cohort was approved by the Ethics Committee of The University of Tokyo Hospital [IRB#: 2406-(8), April 12, 2023]. Due to its retrospective nature, information about this study was provided to all patients through an opt-out process, giving them the option to decline the use of their information.

### Funding

The authors declare that there were no funding sources for this study.

### Data availability

No new data were created or analyzed in this study. Data sharing is not applicable to this article.

### Author contributions

All the authors meet the ICMJE authorship criteria. Conception and design: GT. Administrative support: MS. Provision of study materials or patients: GT, MY, TY, CK, and MS. Collection and assembly of data: GT, MY, TY, and CK. Data analysis and interpretation: GT, MK, and MS. Manuscript writing: All authors. Final approval of the manuscript: All authors. All authors have approved the submitted version of the manuscript and agree to be accountable for all aspects of the work.

### Disclosure statement

The authors have nothing to disclose.

## Supplementary Materials

Supplementary Fig. 1. Study flowchart. (**A**) The analysis population consisted of 398 patients with various lung diseases. (**B**) Overall survival curves for patients with the indicated types of lung disease after they had registered for lung transplantation. IP: interstitial pneumonia

Supplementary Fig. 2. Distribution of CNKS and its cut-off. (**A**) Distribution of CNKS in the 202 patients with IP. (**B**) Receiver-operating characteristic curves were used to evaluate the ability of CNKS to predict the survival of patients with IP after they had registered for LT. CNKS: controlling nutritional status/creatine kinase score; IP: interstitial pneumonia; LT: lung transplantation

Supplementary Fig. 3. Overall survival curves of 398 patients after they had registered for lung transplantation, divided according to their CNKS. CNKS: controlling nutritional status/creatine kinase score

Supplementary Table 1. Characteristics of 398 patients at the time of registration for lung transplantation.
