# Supplementary figures and images for: Musculo-Immuno-Nutritional Score as a Prognostic Marker in Patients with Interstitial Pneumonia Awaiting Lung Transplantation
Source: Ann Thorac Cardiovasc Surg. 2025 Jun 5;31(1):25-00067. doi: 10.5761/atcs.oa.25-00067 (PMC12145925; doi:10.5761/atcs.oa.25-00067)

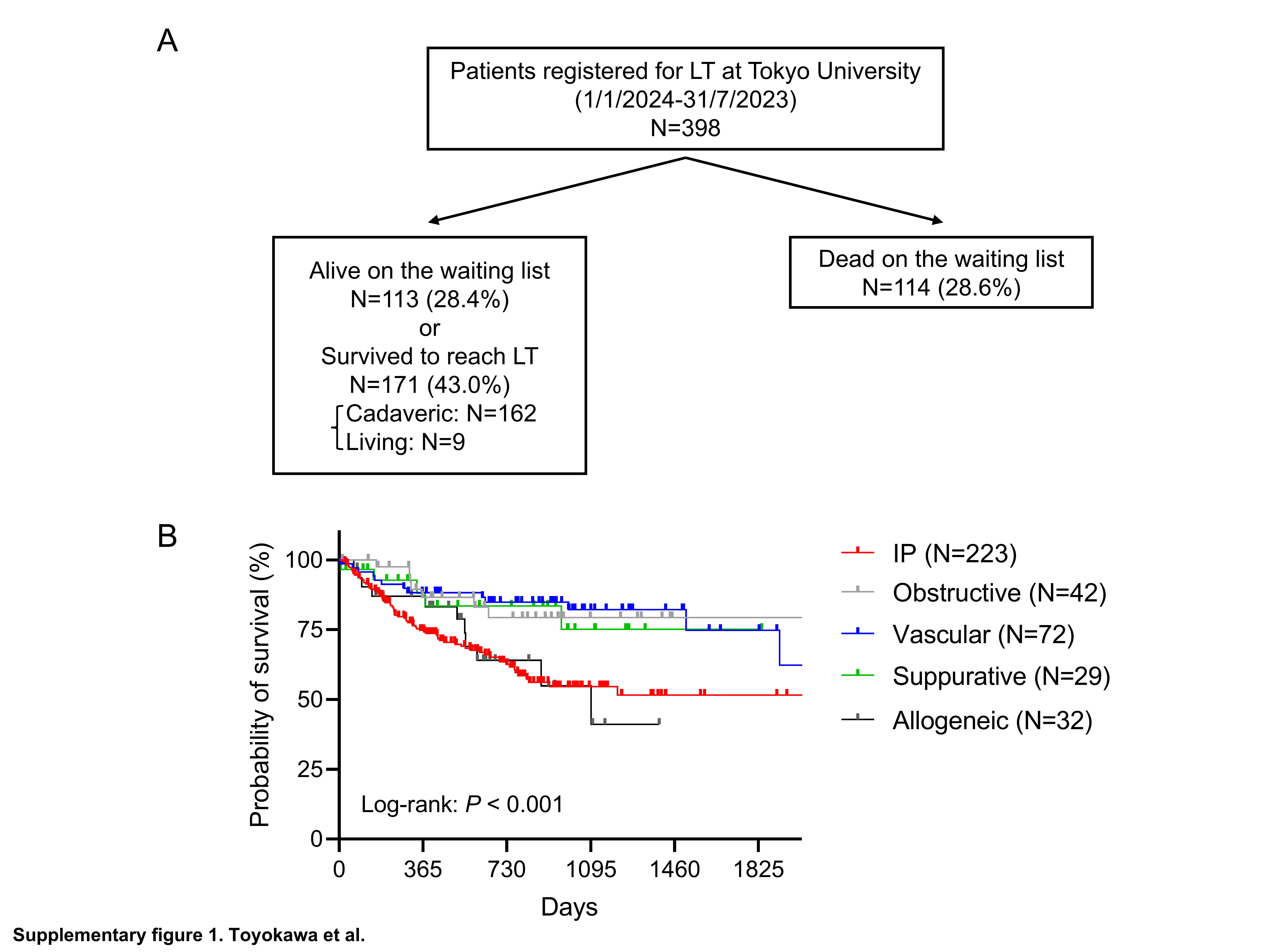

Supplement: Supplementary Fig. 1. [file atcs-31-1-25-00067-s01.TIF]

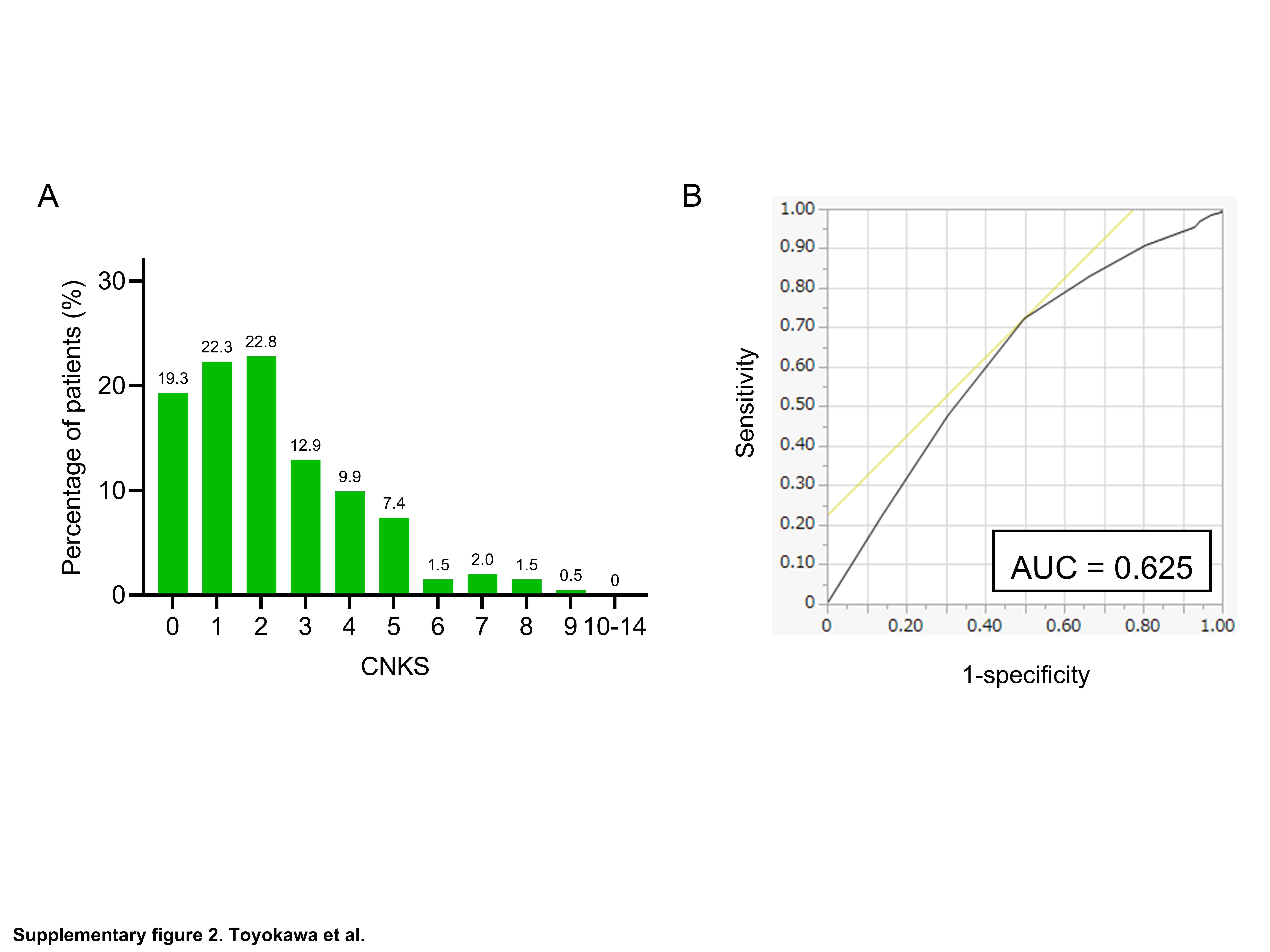

Supplement: Supplementary Fig. 2. [file atcs-31-1-25-00067-s02.TIF]

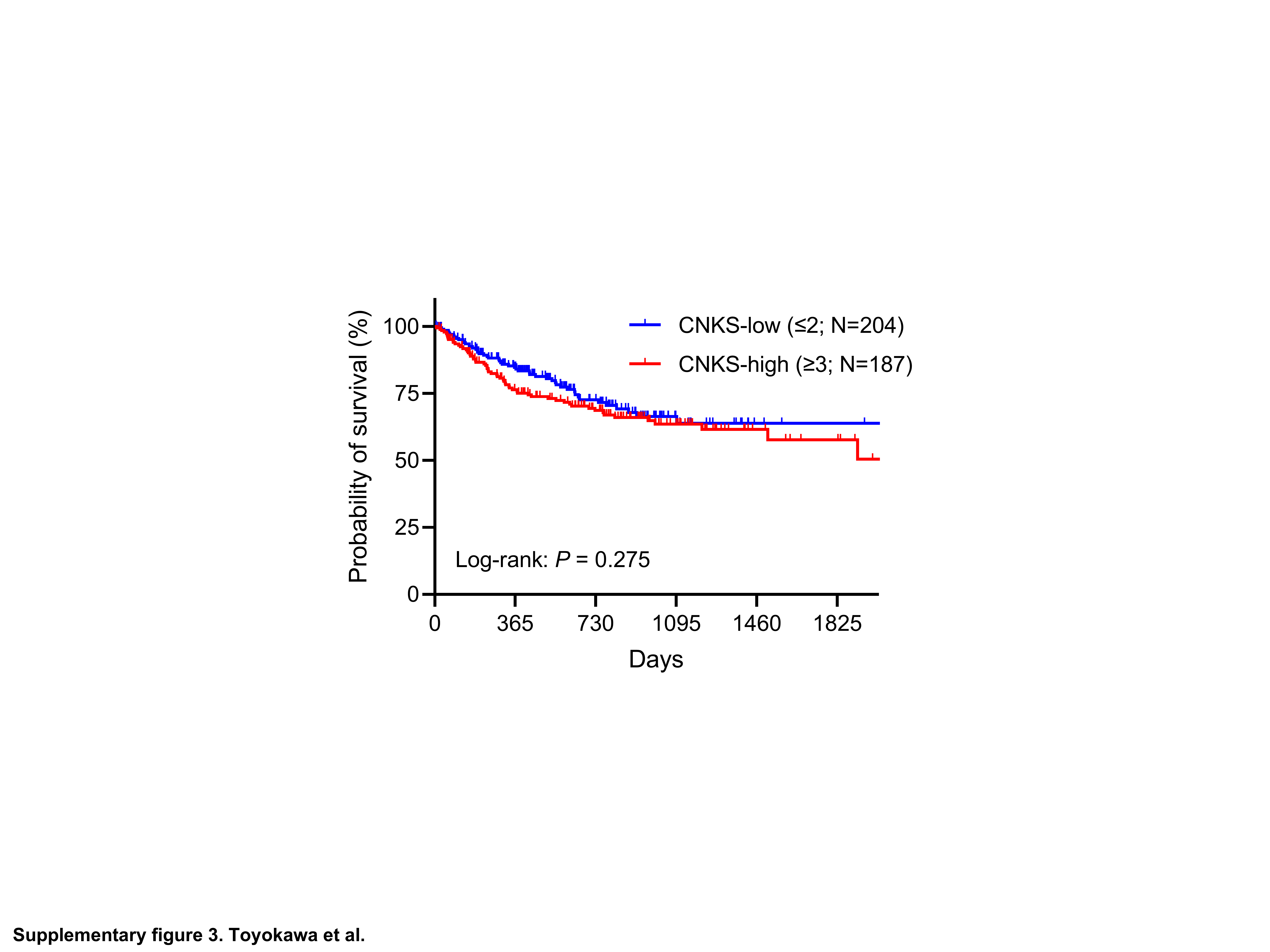

Supplement: Supplementary Fig. 3. [file atcs-31-1-25-00067-s03.TIF]
