# Supplementary material for: Musculo-Immuno-Nutritional Score as a Prognostic Marker in Patients with Interstitial Pneumonia Awaiting Lung Transplantation
Source: Ann Thorac Cardiovasc Surg. 2025 Jun 5;31(1):25-00067. doi: 10.5761/atcs.oa.25-00067 (PMC12145925; doi:10.5761/atcs.oa.25-00067)
Supplement: Supplementary Table 1. [file atcs-31-1-25-00067-s04.pdf]

Supplementary Table 1. Characteristics of 398 patients at the time of registration for lung transplantation.

**Demographic**		**N (%)**
Age at registration (years)*		47 (0–60)
Sex	Female	187 (47.0%)
	Male	211 (53.0%)
Smoking history	Never	210 (52.8%)
	Ex- or current	188 (47.2%)
BMI (kg/m^2)*		19.8 (10.5–34.9)
Blood type	A	157 (39.4%)
	O	107 (27.0%)
	B	98 (24.6%)
	AB	36 (9.0%)
Disease category	IP	223 (56.0%)
	Vascular	72 (18.1%)
	Obstructive	42 (10.6%)
	Suppurative	29 (7.3%)
	Allogeneic	32 (8.0%)
Laboratory data*	Albumin level (g/dL)	4.0 (2.7–4.9)
	Creatine kinase level (IU/L)	72 (8–334)
	Total cholesterol level (mg/dL)	195 (117–357)
	Lymphocyte count ($10^3/\mu L$)	1.5 (0.2–4.3)

BMI, body mass index; IP, interstitial pneumonia.

*: Median value (range)
